# Investigating T-cell-derived extracellular vesicles as biomarkers of disease activity, axonal injury, and disability in multiple sclerosis

**DOI:** 10.1093/cei/uxaf003

**Published:** 2025-01-11

**Authors:** Jennifer L Zagrodnik, Stephanie N Blandford, Neva J Fudge, Shane T Arsenault, Sarah Anthony, Lillian McGrath, Fraser Clift, Mark Stefanelli, Craig S Moore

**Affiliations:** Division of Biomedical Sciences, Faculty of Medicine, Memorial University of Newfoundland, St. John’s, NL, Canada; Division of Biomedical Sciences, Faculty of Medicine, Memorial University of Newfoundland, St. John’s, NL, Canada; Division of Biomedical Sciences, Faculty of Medicine, Memorial University of Newfoundland, St. John’s, NL, Canada; Discipline of Medicine (Neurology), Faculty of Medicine, Memorial University of Newfoundland, St. John’s, NL, Canada; Discipline of Medicine (Neurology), Faculty of Medicine, Memorial University of Newfoundland, St. John’s, NL, Canada; Discipline of Medicine (Neurology), Faculty of Medicine, Memorial University of Newfoundland, St. John’s, NL, Canada; Discipline of Medicine (Neurology), Faculty of Medicine, Memorial University of Newfoundland, St. John’s, NL, Canada; Discipline of Medicine (Neurology), Faculty of Medicine, Memorial University of Newfoundland, St. John’s, NL, Canada; Division of Biomedical Sciences, Faculty of Medicine, Memorial University of Newfoundland, St. John’s, NL, Canada; Discipline of Medicine (Neurology), Faculty of Medicine, Memorial University of Newfoundland, St. John’s, NL, Canada

**Keywords:** multiple sclerosis, extracellular vesicles, biomarkers, T cells, plasma

## Abstract

**Introduction:**

Multiple sclerosis (MS) is a chronic immune-mediated demyelinating disease of the central nervous system, whereby clinical disease activity is primarily monitored by magnetic resonance imaging.

**Methods:**

Given the limitations associated with implementing and acquiring novel and emerging imaging biomarkers in routine clinical practice, the discovery of biofluid biomarkers may offer a more simple and cost-effective measure that would improve accessibility, standardization, and patient care. Extracellular vesicles (EVs) are nanoparticles secreted from cells under both homeostatic and pathological states, and have been recently investigated as biomarkers in MS. The objectives of this study were to longitudinally measure levels of specific immune cell-derived EVs in MS and provide evidence that EV sub-populations may serve as biomarkers of disease activity, axonal injury, and/or clinical disability.

**Results:**

Our results demonstrate that the rate of clinical disability in MS negatively correlates with changes in circulating CD3^+^ EVs within the plasma. Additionally, numbers of CD4^+^ EVs decrease in individuals with increasing pNfL levels overtime whereby the magnitude of the pNfL increase negatively correlates with changes in plasma CD4^+^ and CD8^+^ EVs. Finally, when applying NEDA-3 criteria to define active versus stable disease, individuals with active disease had significantly elevated CD4^+^ and CD8^+^ EVs compared to stable disease.

**Conclusion:**

In summary, the analysis of specific immune cell-derived EV subsets may provide a method to monitor disability accumulation, disease activity, and axonal injury in MS, while also providing insights into the pathophysiology and cellular/molecular mechanisms that influence progression.

## Teaser

Extracellular vesicles (EVs) are small vesicles that are secreted from cells under both physiological and pathological conditions. In multiple sclerosis (MS), individuals experiencing a longitudinal increase in clinical disability, MRI disease activity, and axonal injury had significantly elevated plasma levels of specific T-cell-derived EVs, thereby suggesting the utility of circulating EVs as novel biomarkers in MS.

## Graphical Abstract

**Graphical Abstract:**
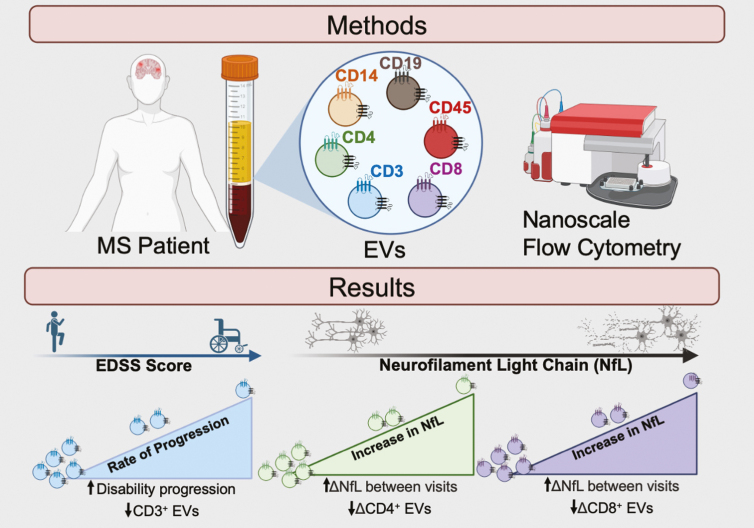

## Introduction

Multiple sclerosis (MS) is a chronic immune-mediated disease of the central nervous system (CNS) that results in neuroinflammation, demyelination, and neuronal injury [1]. The expanded disability status scale (EDSS) is currently the clinical standard for measuring both clinical disability and disease progression in MS patients [2]. The limitations and caveats of the EDSS have been previously and widely acknowledged given scores are primarily and heavily reliant on walking ability with less emphasis and an inadequate assessment of cognition, mood, fatigue, and bowel/bladder function [2, 3]. As such, together with the highly heterogenous nature of MS, clinicians’ and researchers’ abilities to reliably and accurately monitor, measure, and predict disease activity remains challenging [4].

A biomarker is an objective measure that is indicative of health status or a pathological state [5]. In MS, magnetic resonance imaging (MRI) is the primary biomarker that is used at diagnosis and when monitoring disease activity, progression, and treatment response [4–6]. The application of putative biofluid-biomarkers in MS provides the possibility for a more objective, standardized, cost-effective, time-effective, and minimally invasive means of measuring various disease parameters.

Neurofilament light chain (NfL) is an intermediate filament that functions in the structural integrity of axons and is detectable in the cerebrospinal fluid (CSF) and blood following axonal injury [7, 8]. Increased levels of NfL have been measured in nearly all neurodegenerative diseases, traumatic head injury, and are also associated with natural aging [7–9]. Within the context of MS, elevated NfL levels are associated with increased clinical disability worsening, relapse activity, and the number and volume of demyelinated lesions [10–12]. However, the lack of specificity and the several confounding variables that can influence NfL levels in humans warrant further investigation into novel biofluid biomarkers in MS [8].

The term extracellular vesicles (EVs) encompasses all small (30–1000nm) membrane-bound vesicles (e.g. exosomes, microvesicles, apoptotic bodies) that are secreted from all cell types under both homeostatic and pathological conditions [13, 14]. EVs contain active biomolecules (e.g. nucleic acids, proteins, lipids) and play a pivotal role in intercellular communication [15]. Together, both the surface expression of various tetraspanin molecules and the accompanying molecular cargo are often representative of an EV’s cell-of-origin and can be used to quantify and phenotype EV populations from individual cells of interest [16, 17].

In MS, EVs within various biofluids have been proposed as potential biomarkers of disease activity, progression, and treatment response [17, 18]. Specifically, we have previously demonstrated that EVs derived from specific immune cell subsets, including several types of lymphocytes and myeloid cells, are elevated within the plasma of mildly disabled and DMT naïve RRMS patients compared to their age- and sex-matched counterparts [19]. Additionally, the associated changes in EV sub-populations were not correlated with changes in their respective parent immune cells, suggesting that the detection of EVs within plasma is likely representative of immune cell activity/status and not due to the overall abundance of individual immune cells within circulation [19].

The objective of this research is to provide insights into whether longitudinal measures of specific immune cell-derived EVs in MS can serve as biomarkers of clinical disability, axonal injury, and/or disease activity. Using both cross-sectional and longitudinal analyses, our results demonstrate that levels of plasma EV sub-populations secreted from T cells (CD4^+^ & CD8^+^) are significantly altered in the plasma of active versus stable MS. Furthermore, the magnitude of longitudinal changes (*i.e.* delta) in disability scores and neurofilament light chain (NfL) levels of individual patients correlated with changes in EV sub-populations overtime. Finally, this study also highlights the importance of intra-patient and longitudinal analyses when investigating EVs as potential biomarkers in MS.

## Methods and materials

### Sex as a biological variable

Our study examined male and female human participants with similar findings reported for both sexes.

### Human sample collection and preparation

Protocols and experiments were approved by the Newfoundland Health Research Ethics Board. Written informed consent was obtained from all participants prior to study initiation. MS patients were diagnosed according to 2017 McDonald criteria [6] and recruited through the Health Research Innovation Team in Multiple Sclerosis (HITMS), an MS patient registry and biorepository at Memorial University of Newfoundland, St. John’s, Canada between February 2016 and March 2024.

A cross-sectional relapsing-remitting MS (RRMS) cohort was selected to analyze EV concentration, EV size, and plasma levels of various immune cell-derived EVs with EDSS, disease activity, and pNfL levels (Table 1). A second longitudinal RRMS cohort (Table 2) was studied based on individuals having multiple longitudinal follow-up visits and had either an increase in the Expanded Disability Status Scale (EDSS) score of 1.0 or greater (subcohort #2A), or an increase in plasma neurofilament light chain of ≥1.0 pg/ml (subcohort #2B). Exclusion criteria included individuals changing a disease-modifying therapy (DMT) within the first year following the blood draw, as research has shown that DMTs (e.g. natalizumab) can significantly alter peripheral PMBCs and immune cell-derived EVs [20, 21].

**Table 1:** clinical demographics for cross sectional analyses of EDSS, pNfL, and disease activity in individuals with RRMS

Cohorts	EDSS cross sectional cohort	pNfL cross sectional cohort	Disease activity cross sectional cohort	
Groups			*Stable*	*Active*
No. of participants	26	26	9	10
Age (mean ± SD)	50 ± 10.81	49 ± 10.88	49 ± 4.50	43 ± 13.09
Sex [# (%)]				
Female	18 (69%)	18 (69%)	6 (67%)	7 (70%)
Male	8 (31%)	8 (31%)	3 (33%)	3 (30%)
EDSS (mean ± SD; range	2.21 ± 1.67; 0.0–6.0	1.25 ± 1.45; 0.0–5.5	0.72 ± 1.15; 0.0–3.0	2.45 ± 2.17; 0.0–6.5
pNfL (pg/mL) (mean ± SD; range)	11.36 ± 6.43; 3.71–29.70	10.87 ± 7.78; 2.19–30.35	6.57 ± 2.95; 4.91–12.38	15.01 ± 8.04; 5.14–29.70
pNfL *Z*-score (mean ± SD; range)	+0.43 ± 1.31; −1.31 to + 2.75	−1.04 ± 0.90; −2.18 to + 2.75	−0.80 ± 1.34; −2.81 to + 0.84	+1.63 ± 0.94;+0.18 to + 2.75
Disease duration (years; mean ± SD)	14.92 ± 6.28	13.44 ± 5.75	14.11 ± 6.39	12.2 ± 7.1
Disease modifying therapies [# (%)]				
Dimethyl fumarate	5 (19%)	7 (27%)	4 (44%)	1 (10%)
Glatiramer acetate	4 (15%)	4 (15%)	1 (11%)	2 (20%)
Fingolimod	8 (31%)	7 (27%)	3 (34%)	4 (40%)
Teriflunomide	4 (15%)	4 (15%)		
Interferon beta-1a	3 (12%)	3 (12%)	1 (11%)	1 (10%)
DMT free	2 (8%)	1 (4%)	-	2 (20%)

**Table 2:** clinical demographics for longitudinal analyses of EDSS and pNfL in individuals with RRMS

Cohorts	Longitudinal EDSS cohort		Longitudinal pNfL cohort	
Groups	*Visit 1*	*Visit 2*	*Visit 1*	*Visit 2*
No. of participants	17	17	16	16
Age (year; average ± SD)	45 ± 12.0	48 ± 12.11	49 ± 11.00	51 ± 10.75
Sex [# (%)]				
Female	11 (65%)	11 (65%)	12 (75%)	12 (75%)
Male	6 (35%)	6 (35%)	4 (25%)	4 (25%)
EDSS (mean ± SD; range)	1.26 ± 1.56; 0.0–5.5	2.88 ± 1.67; 1.0–6.5	1.225 ± 1.50; 0.0–5.5	2.375 ± 1.77; 1.0–6.5
pNfL (pg/mL) (mean ± SD; range)	8.76 ± 4.29; 2.19–18.99	11.51 ± 6.17; 5.14–29.70	9.02 ± 5.20; 2.19–20.33	13.74 ± 7.31; 5.40–30.35
pNfL Z-score (mean ± SD; range)	+0.20 ± 1.43; −2.81 to + 2.2	+0.75 ± 1.14; −0.55 to + 2.75	−0.14 ± 1.37; −2.81 to + 2.10	+0.87 ± 1.20; −1.51 to + 2.75
Disease duration (years; mean ± SD)	14.47 ± 6.56	17.00 ± 6.70	15.31 ± 6.06	18.06 ± 5.88
Disease modifying therapies (DMTs)				
Dimethyl fumarate	7 (41%)	4 (23%)	4 (25%)	2 (13%)
Glatiramer Acetate	2 (12%)	2 (12%)	3 (18%)	3 (18%)
Fingolimod	4 (23%)	4 (23%)	5 (31%)	5 (31%)
Teriflunomide	2 (12%)	3 (18%)	2 (13%)	3 (18%)
Interferon beta-1a	2 (12%)	2 (12%)	2 (13%)	2 (13%)
DMT-free		2 (12%)		1 (6%)

Magnetic resonance imaging (MRI) scans were performed as per routine clinical assessment and evaluated by board-certified neuroradiologists. Classification of a patient’s disease activity was based on the no evidence of disease activity-3 (NEDA-3) criteria [22, 23]. A stable categorization was defined as the absence of evidence of: (i) clinical relapses, (ii) new or enlargement of T2-weighted and/or gadolinium-enhanced lesions, and (iii) disease progression, which was defined as an increase of at least 1.5, 1.0, or 0.5 or greater for EDSS scores of 0, 1.0–5.0, and greater than 5.5, respectively [24, 25]. Individuals categorized as active were defined based on the failure to meet the NEDA-3 criteria, which included the occurrence of any combination of the following ±12 months between blood sample collection: (i) an increase or enlargement of ≥ one brain and/or spinal cord lesions observed on T2-weighted MRI, (ii) evidence of clinical relapse, and/or (iii) subsequent increases in EDSS relevant to their baseline score [24, 26]. All patients were selected based on the previously described criteria and sample availability.

Human blood was obtained *via* vein-puncture into BD Vacutainer K2 EDTA tubes (Becton Dickson: 366643). To collect plasma, the tubes were centrifuged at 300 x g for 10 minutes and aliquoted into cryogenic vials (Thermofisher: 5000-1012), stored at −80°C for approximately 24 hours, and then relocated to liquid nitrogen for long-term storage. For PBMC isolation, 20 ml of whole blood and 15 ml of PBS were added to 50 ml SepMate^TM^ tubes (StemCell Technology: 85450) containing 15 ml of Ficoll (Cytiva: 17144003) and centrifuged at 12 000 x g for 10 minutes with the brake on. The pelleted cells on the side of the tube were carefully removed and collected. The cells were then transferred to a new 50 ml conical tube (Fisher Scientific:14-432-22), centrifuged at 300 x g for 5 minutes, decanted, and resuspended in PBS. Isolated PBMCs were resuspended in a freezing media solution comprised of 70% fetal bovine serum (FBS) (Fisher: FB12999102), 10% Roswell Park Memorial Institute media (RPMI) (ThermoFisher: 31800-022), and 20% dimethyl sulfoxide (DMSO) (Fisher Scientific: BP-231-100) and stored in liquid nitrogen.

### EV flow cytometry

All experiments were previously established, reviewed, and published in accordance with the MIFlowCyt-EV framework [19, 27]. Plasma EVs were quantified using violet (405 nm) side scatter (SCC) flow cytometry on a CytoFLEX^TM^ (Beckman Coulter, Indianapolis, USA) as previously described [19]. Briefly, cryopreserved plasma samples were thawed at room temperature (RT) and centrifuged at 2000 x g for 20 minutes to remove cell debris. BD Pharmingen™ PE mouse anti-human antibodies for CD3 (BD Bioscience: 555340), CD4 (BD Bioscience: 555347), CD8 (BD Bioscience: 555635), CD14 (BD Bioscience: 555398), CD19 (BD Bioscience: 555413), CD45 (BD Bioscience: 555483), and CD9 (BD Bioscience: 555372) were centrifuged at 12 500 x g for 10 minutes. Each antibody was then added to an individually prepared sample containing 2.5 μl of plasma diluted in 2.5 μl of 0.1 µm sterile-filtered PBS (Cytiva: SH30256.10) and stained in the dark for 1 hour at RT. After 1 hour, samples were further diluted to bring the total volume to 25 μl using filtered PBS. Subsequently, the samples were diluted 1:400 in filtered PBS and the data was acquired for 10 minutes at a flow rate of 10 µl/min using a CytoFLEX^TM^ (Beckman Coulter, Indianapolis, USA). Gating and analysis were performed using FlowJo (FlowJo, LLC, Ashland, OR) software.

### PBMC flow cytometry

Cryopreserved PBMCs from RRMS patients were quickly thawed from liquid nitrogen, resuspended in cold RPMI (ThermoFisher: 31800-022), and centrifuged for 10 minutes at 300 x g. Cells were then counted and resuspended at a density of 500 000 cells/ml in a washing solution made of 1% fetal bovine serum (FBS) (Fisher: FB12999102) in PBS with 2 nM EDTA and 2 nM sodium azide. Cells were centrifuged at 300 x g for 5 minutes, decanted, and resuspended in 100 μl of the washing solution. The cell suspension was then added to a DURAclone IM Phenotyping BASIC tube (Beckman Coulter: B53309), mixed, and incubated at 4°C for 30 minutes. After incubation, 3 ml of the washing solution was added to the tube and centrifuged at 300 x g for 5 minutes. The cells were decanted and resuspended in 100 µl of the fixative agents composed of 2% paraformaldehyde. 100 000 events were acquired for each sample using the CytoFLEX^TM^ (Beckman Coulter, Indianapolis, USA) and analyzed using FlowJo software.

### Nanoparticle tracking analysis

The concentration and size of nanoparticles within plasma were measured using the NanoSight NS3000 (Malvern Panalytical, Malvern, UK). Plasma samples from RRMS patients were diluted 1:1000 in 0.1 μm filtered PBS. The diluted sample was placed into a 1 ml plastic syringe (Fisher Scientific: 14-817-25) and was run through the NanoSight^TM^ using the automatic pump set to 25.0. All samples were run on identical camera settings, which included: Laser Type: Blue488, Camera Level: 15:0 Camera gain: 1.0, slider shutter: 1232, Slider Gain: 219, Frame rate: 25.0 FPS, Temperature: 25°C, Detect Threshold: 10.0, Blur size: auto, Max Jump Distance: auto.

### SiMoA Assay for plasma neurofilament light chain

Plasma neurofilament light chain (pNfL) was measured using the Quanterix Simoa® NfL assay (Quantrix, MA, USA). Selected RRMS plasma samples were aliquoted and shipped on dry ice (Toronto, Ontario), and stored at −80°C until subsequent analysis, averaging the duplicates, following the manufacturer’s instructions. The corresponding Z-score of pNfL levels was based on the mean of pNfL values from age and BMI-matched control populations, and were used to account for the influence of both factors on the levels of circulating pNfL in the selected cohort. pNfL Z-scores were obtained using an internet-based app: https://shiny.dkfbasel.ch/baselnflreference/

### Statistical analysis

All statistical analyses were performed using Prism9 (GraphPad Software Inc. San Diego, CA, USA). Statistical outliers were identified using the extreme studentized deviate method (also known as Grubbs test). Correlations of NTA data of concentration and size of EVs and flow cytometry data from immunophenotyped EVs vs. age, disease duration, EDSS, and pNfL were analyzed using Pearson *r* correlations. All statistical analyses included corrections for multiple comparisons using Holm–Sidak method when necessary. Data analyzed using unpaired t-tests included disease activity groups (stable vs. active). Longitudinal data of individuals with MS were analyzed using paired *t*-tests. PBMC data were normally distributed and paired *t*-tests, unpaired *t*-tests and Pearson correlations were used to analyze PBMCs in relation to age, disease duration, disease activity, EV concentration, EV size, and populations of immune cell-derived EVs.

## Results

### Circulating total plasma EVs can be quantified using nanoscale flow cytometry and are correlated with clinical disability in RRMS

**Figure 1:**
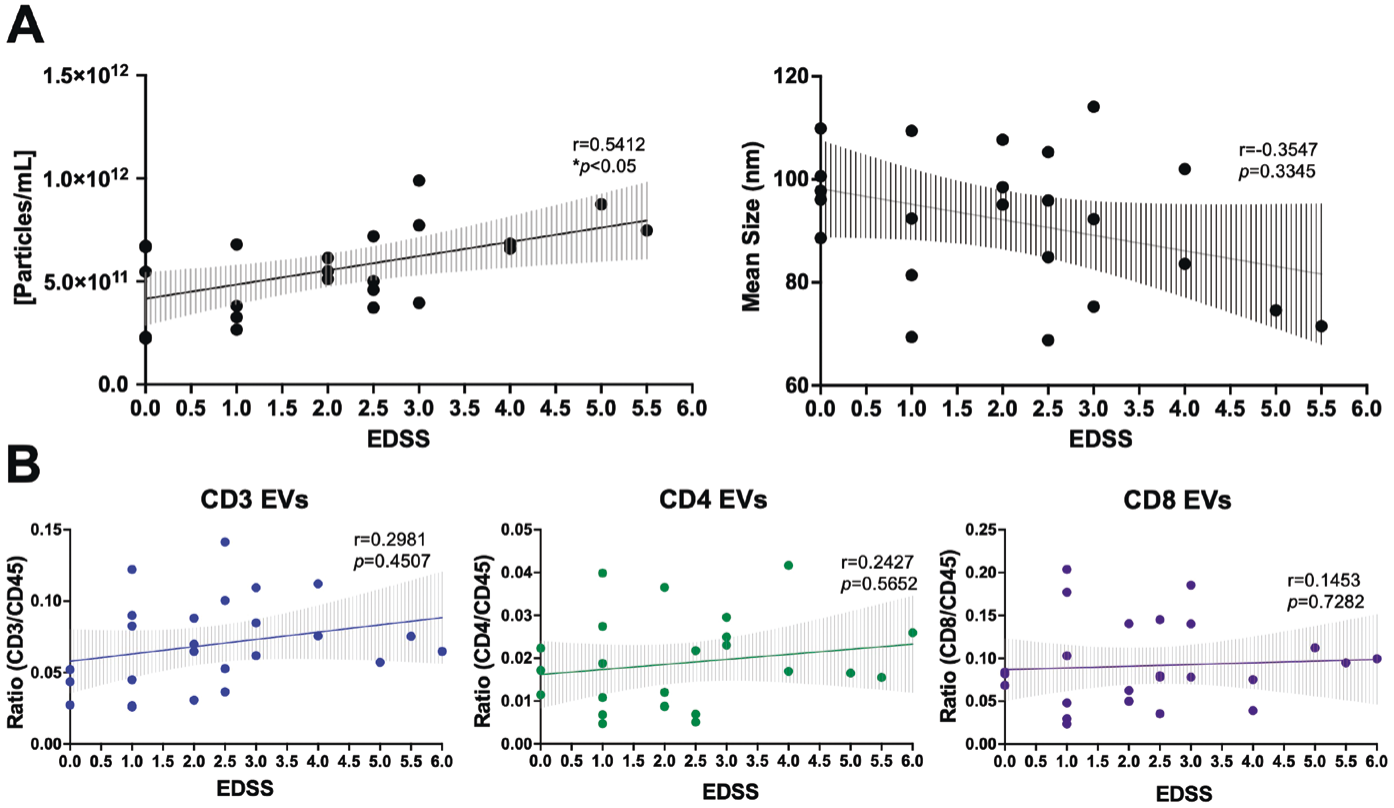
plasma EV concentration is correlated with clinical disability in RRMS. **(A)** Plasma EV concentration is positively correlated with EDSS score (*r* = 0.5412, *P* = 0.0304, *n* = 23). No correlation between mean EV size and EDSS score was observed. **(B)** EDSS does not correlate with levels of specific immune cell-derived EVs within the plasma of individuals with RRMS (*n* = 26). Error bars represent mean ± SEM

Total levels of circulating leukocyte-derived EVs within the plasma of individuals with RRMS was quantified using V-SSC flow cytometry as previous [19] (Supplementary Fig. S1A). Briefly, diluted plasma was stained with PE-labeled antibodies (CD3, CD4, CD8, CD14, CD19, CD45, or CD9) with a gating strategy that was based on an unstained EV sample and expression of CD9 (Supplementary Fig. S1A). Successful EV isolations were confirmed using electron microscopy, western blot, and nanoparticle tracking analysis (NTA, Supplementary Fig. S1B–D). Using nanoscale flow cytometry, the concentration of plasma EVs positively correlated with clinical disability, as measured by EDSS scores (Fig. 1A; *P* = 0.0304). Neither the mean size of plasma EVs, nor the numbers of individual immune cell-derived EV subsets, were correlated with EDSS scores (Fig. 1A&B, Supplementary Fig. 2). In a cross-sectional analysis, potential relationships among patient sex, disease duration, EV concentration, EV size, and plasma levels of specific immune cell-derived EVs were also investigated. No significant sex differences were observed between these variables (Supplementary Figs. S3 and 4).

### The magnitude of decrease in plasma-derived CD3^+^ EVs is associated with a more rapid disability progression

**Figure 2:**
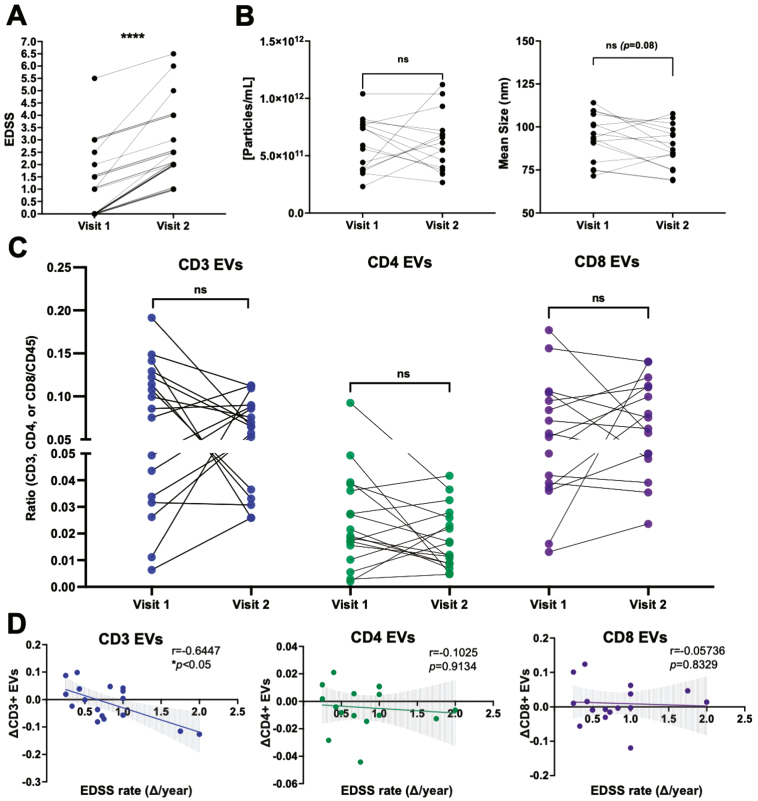
the magnitude of decrease in circulating CD3^+^ EVs is correlated with greater disability progression. **(A)** Longitudinal plasma samples were analyzed from individuals with RRMS who experienced an increase in EDSS between two time points (*n* = 17). **(B)** The concentration and mean size of plasma EVs does not significantly change over time in individuals with RRMS who experience an increase in disability (*n* = 16). **(C)** No differences in the levels of CD3^+^, CD4^+^, or CD8^+^ EVs were observed in this cohort, however the magnitude of decrease in CD3^+^ EVs negatively correlated with the rate of clinical disability progression (**D**) (*r* = −0.644, *P *= 0.0155, *n* = 17). Error bars represent mean ± SEM

To investigate potential longitudinal relationships among clinical disability and EV concentration, EV size, and plasma levels of specific immune cell-derived EVs, plasma samples from individuals with RRMS that experienced an increase in EDSS between two time-points were selected (≥1.0 point; Fig. 2A). The rate of disability was determined based on the change in EDSS and the time duration between the two time-points selected (i.e. ΔEDSS/number of years) [28]. In individuals that experienced an increase in clinical disability, no significant differences in the concentration or mean size of plasma EVs were observed (Fig. 2B), nor were there any significant differences in the levels of immune cell-derived EVs (Fig. 2C, Supplementary Fig. S5A). However, when investigating potential relationships between the levels of circulating immune cell-derived EVs and the rate of disability progression, the magnitude of decrease in plasma-derived CD3^+^ EVs negatively correlated with the rate of change in clinical disability (i.e. individuals experiencing the most rapid disability progression had the greatest decrease in CD3^+^ EVs (Fig. 2D; *P* = 0.0155). No correlations were observed in the other immune cell-derived EV subsets (CD14, CD19, total CD45; Fig. 2D, Supplementary Fig. 5B).

### The magnitude of decrease in plasma-derived CD4^+^ and CD8^+^ EVs correlates with the magnitude of increase in pNfL levels in RRMS

**Figure 3:**
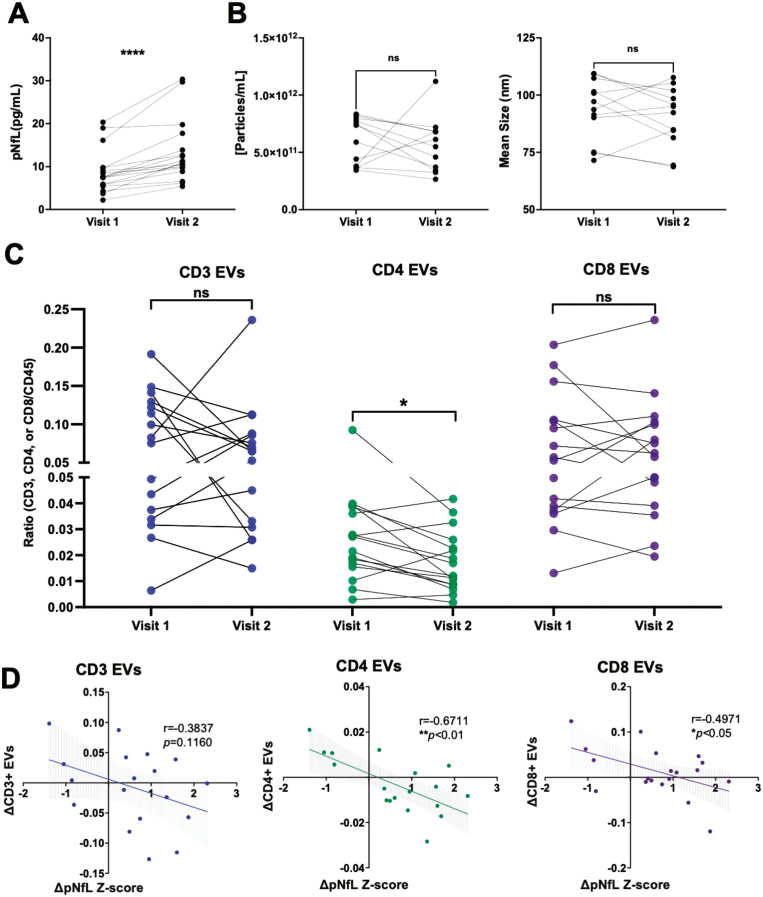
the magnitude of decrease in circulating CD4^+^ and CD8^+^ EVs is correlated with the magnitude of increase in pNfL in RRMS. **(A)** Longitudinal plasma samples were analyzed from individuals with RRMS who experienced an increase in NfL levels (≥1 pg/ml) between two time points (*n* = 16). (**B**) The concentration and mean size of plasma EVs does not significantly change over time in individuals with RRMS who experience an increase in NfL levels (*n* = 16). (**C**) A significant decrease in the levels of CD4^+^ EVs were measured in individuals with RRMS who experience an increase in NfL levels while the magnitude of decrease in CD4^+^ and CD8^+^ EVs negatively correlated with the change in NfL Z-scores (**D**). Error bars represent mean ± SEM

To investigate potential relationships between axonal injury (measured by pNfL levels) and EV concentration, EV size, and plasma levels of specific immune cell-derived EV subsets, pNfL Z-scores were used [29]. In a cross-sectional analysis, neither the concentration, size of plasma-derived EVs, nor levels of immune cell-derived EV subsets were correlated with pNfL Z-scores (Supplementary Fig. S6). To investigate potential longitudinal relationships between axonal injury and EVs, samples from individuals with RRMS that experienced an increase in pNfL levels between two time-points were selected (≥1.0 pg/ml; Fig. 3A). In individuals with increasing pNfL levels, similar to individuals with increasing clinical disability, no significant differences in the concentration or size of EVs were observed (Fig. 3B). In contrast to individuals with increasing disability, a significant decrease in the levels of CD4^+^ EVs was measured in patients with increasing pNfL levels (Fig. 3C; *P* = 0.0260). Additionally, the magnitude of decrease in plasma-derived CD4^+^ (Fig. 3D; *P* = 0.0069) and CD8^+^ (Fig. 3D; *P* = 0.0303) negatively correlated with the magnitude of increase in pNfL Z-scores (i.e. the greater the increase in pNfL, the greater the decrease in circulating CD4^+^ and CD8^+^ EVs). No differences or correlations were observed in the other immune cell-derived EV subsets (CD14, CD19, total CD45; Supplementary Fig. S7).

### Levels of CD4^+^ and CD3^+^ EVs are significantly increased in individuals with RRMS active disease, compared to RRMS stable, despite no change in numbers of their respective immune cells

**Figure 4:**
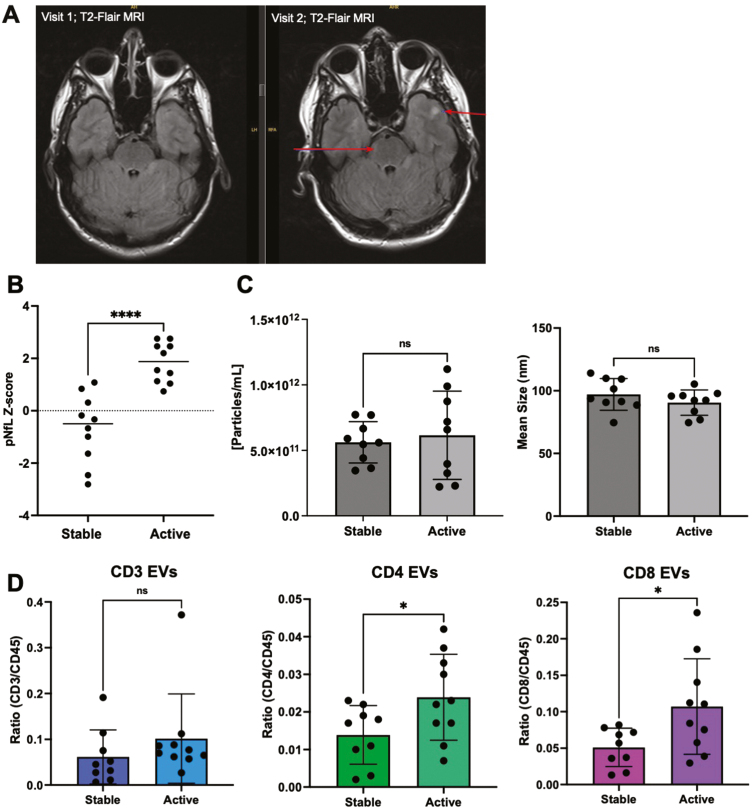
levels of CD4^+^ and CD8^+^ EVs are significantly increased in individuals with RRMS active disease. **(A)** Representative image of increased number of lesions (arrows) in an individual with RRMS. The formation of two new lesions was detected on T2-FLAIR axial MRI after eight months. **(B)** Individuals deemed active (failed to meet NEDA-3 criteria) had significantly greater levels of pNfL compared to stable individuals with RRMS (*p* < 0.0001; n = 9–10). **(C)** No significant differences in the concentration or mean size of plasma EVs were observed in active MS vs stable MS (*n* = 9–10). **(D)** In plasma, levels of CD4^+^ and CD8^+^ EVs were significantly greater in active MS vs stable MS. *P < 0.05. Error bars represent mean ± SEM.

To investigate potential relationships between disease activity and EV concentration, EV size, and plasma levels of immune cell-derived EV subsets, individuals with RRMS were defined as either clinically stable or clinically active based on NEDA-3 criteria [23], one of which is no increases in the size or number of T2-weighted lesions (Fig. 4A). RRMS active individuals had significantly higher pNfL Z-scores compared to RRMS stable (Fig. 4B; *P *< 0.0001), however, no differences in EV concentration or size were observed (Fig. 4C). Plasma levels of CD4^+^ and CD8^+^ EVs were significantly greater in active MS (Fig. 4D; *P* = 0.047 and 0.0232, respectively) compared to individuals with stable disease. No differences were observed in the other immune cell-derived EV subsets (CD14, CD19, total CD45; Supplementary Fig. S8). Circulating immune cell subsets were also measured using flow cytometry to investigate potential relationships between the numbers of circulating peripheral immune cells and EVs expressing similar surface markers (Supplementary Fig. S9A). All PBMC samples analyzed were from a majority subset of the individuals included in the EV analysis (selected based on sample availability). Cross-sectionally, the number of individual immune cell subsets (CD3, CD4, CD8, CD14, and CD19) did not correlate with levels of their corresponding EVs (data not shown). Furthermore, no differences in the numbers of individual immune cells were observed between active MS vs. stable MS (Supplementary Fig. S9B). In an intra-patient longitudinal analysis, no significant differences in the numbers of immune cell subsets within individuals that experienced an increase in clinical disability and/or pNfL were observed (Supplementary Fig. S9C). Finally, changes in the numbers of immune cell subsets were not correlated with changes in levels of respective EV counterparts (Supplementary Fig. S9D).

## Discussion

Using both cross-sectional and longitudinal analyses, this study aimed to investigate the levels and phenotypic properties of various immune cell-derived EVs for the purpose of identifying novel biofluid biomarkers in MS. While both types of analyses can provide valuable insights, longitudinal data is essential for understanding the heterogeneous nature of the MS disease course and provides an important assessment of an individual patient’s disease course over time [30]. Herein, we demonstrated that within a RRMS patient cohort, T-cell-derived EV subsets were significantly altered over time and were associated with clinical disability, axonal injury, and disease activity. To the best of our knowledge, this is the first report that has investigated longitudinal changes in immune cell-derived EV populations in MS, which provides insights into both novel pathophysiological mechanisms and the potential utility of EVs to be used as blood-derived biomarkers in neuroinflammatory diseases.

At present, the EDSS is the gold standard measure of disability in MS and is widely used by clinicians worldwide. However, it is also widely acknowledged that EDSS has limited longitudinal applications as it is lacking in its predictive value and representation of cellular and molecular activity occurring during periods of disease activity and remission [2, 3]. Additionally, the subjective nature of the neurological exam creates inadequate sensitivity to detect subtle degrees of change in disability levels [2, 3]. In attempts to address these limitations, supplementary measures have been adopted, including tests used to measure physicality and cognition (e.g. Nine-Hole Peg Test, Symbol Digit Modalities Test, radiological biomarkers, and fluid biomarkers [3]).

While a biomarker may not always be specific to a particular disease, it is important to investigate its levels in the context of different disease parameters, as it may provide insight(s) into more specific measures, such as clinical disability and/or disease activity in the case of MS. In previous research that has measured plasma EV concentration in relation to diagnostic and disease activity markers in MS, no significant differences between MS and age- and sex-matched controls have been observed [18, 19]. In these studies, both MS cohorts were comprised of relatively low- to moderate-disability with a median of 1.5 and 2.0, respectively [18, 19]. In the current study, our cohort included a wider range of EDSS scores (0–6.5), which allowed us to explore a relationship with disability. Using a cross-sectional analysis, we observed that the concentration of circulating plasma EVs correlated with EDSS scores (Fig. 1A). EV cargo has also been investigated in the context of clinical disability, whereby levels of myelin basic protein inside serum-derived oligodendrocyte-derived EVs has been positively correlated with EDSS scores [31]. In contrast, an additional study demonstrated that levels of myelin oligodendrocyte glycoprotein in serum-derived EVs negatively correlated with EDSS [32]. While this relationship between EV cargo and disability may be dependent on cell-of-origin, our results are suggestive of a simpler metric to monitor disability compared to having to analyze cell-specific EV cargo.

When analyzing various immune cell-derived EV sub-populations cross-sectionally, no significant correlations were observed between the levels of EVs and EDSS (Fig. 1B & Supplementary Fig. S2). However, when assessing longitudinal changes of EVs within individuals, we observed a significant negative correlation with the change in the levels of circulating CD3 + EVs and the rate of clinical disability progression (ΔEDSS/year) (Fig. 2D). Here, individuals with a greater decrease in circulating CD3 + EVs experienced a more rapid increase in their EDSS score. As EVs readily cross the BBB [33], we postulate that CD3 + EVs may indeed be migrating into the CNS and potentially contributing to a pathophysiology that manifests in worsening disability. In line with this hypothesis, previous findings have demonstrated that the presence of plasma-derived EVs within the CNS resulted in increased disease burden and clinical scores in the EAE model [34]. Alternatively, a decrease in circulating CD3 + EVs over time (as patients progress) could be suggestive of less T-cell mediated inflammation that can accompany progression. While there are numerous EV sub-populations that are derived from various cells-of-origin within the plasma, to our knowledge, the functional influence of T-cell derived EVs on CNS cells has yet to be explored. Future studies aimed at assessing the relationships and ratios of immune cell-derived EVs within the plasma versus cerebrospinal fluid (CSF) remains a focus of ongoing studies. While this current study was limited to CSF sample availability, it is important for future studies to determine the presence and levels of specific immune cell-derived EVs in the CNS, as well as measuring any functional effects that these EVs may have on cells within the CNS. Lastly, while plasma EVs were shown to be more abundant in individuals with a higher EDSS score, the decrease observed in CD3 + EVs longitudinally can be explained given lymphocyte-derived EVs make up only a small fraction of total plasma EVs and that their concentration does not correlate with EDSS cross-sectionally (Fig. 1B). The overall increase in plasma EVs may also be the result of increased EV release from different cellular sources (including cells of the CNS, platelets, and endothelial cells), which has been previously documented in MS [35, 36].

To explore whether the CD3^+^ EVs versus EDSS correlation was unique to clinical progression, we also explored EVs in the context of sex, disease duration, and age. No significant differences were noted between males and females when analyzing the plasma EV concentration, size, and levels of specific immune cell-derived EV sub-populations (Supplementary Fig. S3). This result may be related to the specificity of EVs being measured since it was previously reported that CNS endothelial cell-derived EVs were more abundant in the plasma of males compared to females with MS [36].

The noted change in CD3^+^ EVs within the context of disability progression was further expanded upon by analyzing EV levels in the context of axonal injury *via* measuring plasma neurofilament light chain (pNfL) levels. pNfL levels have been suggested as a direct measure of axonal injury and has been extensively investigated in MS as a promising biomarker of disease activity and treatment response [12]. Previously in MS, CD107a+ EVs within plasma (suggestive of CNS origin) have been reported to be correlated with serum NfL levels [37]. Interestingly, NfL has also been investigated as molecular cargo within EVs themselves with EV-NfL levels being reportedly increased following traumatic brain injury and in individuals diagnosed with Parkinson disease who experience advanced motor difficulties [38, 39]. Since plasma NfL levels are influenced by age and BMI, we used the web-based calculator produced by Benkert et al., which provides Z-scores of NfL based on the age- and BMI-matched averages from a healthy control population; the use of Z-scores aids in a clearer interpretation of any MS-related changes to NfL levels in our cohort [8]. In MS, elevated serum NfL Z-scores are associated with the time since relapse activity, numbers of contrasting-enhancing lesions, and T2-lesion volume [12]. Additionally, serum NfL Z-scores of ≥1.25 showed an increased likelihood for NEDA-3 patients to display clinical and/or radiological activity over the subsequent year [12]. In a cross-sectional analysis, we did not observe any correlations between pNfL Z-scores and plasma EV concentration, EV size, or the levels of various leukocyte-derived EVs (Fig. 3). In contrast, however, a longitudinal analysis demonstrated that numbers of plasma-derived CD4^+^ EVs decrease as pNfL levels increase (Fig. 3C). Furthermore, the magnitude of change in circulating CD4^+^ and CD8^+^ EVs negatively correlated with magnitude of change in pNfL (i.e. the larger the increase in pNfL, the larger the decrease in CD4^+^ and CD8^+^ EVs (Fig. 3D)). The decrease in these EV populations may suggest that T-lymphocyte-derived EVs are being trafficked from the periphery to the CNS following injury. Consistent with this hypothesis, levels of T-cell derived EVs in MS patients, specifically CD4^+^CCR3^+^ and CD4^+^CCR5+, are elevated in the CSF of individuals with Gad+ lesions compared to Gad− lesions [40]. Previous research has also highlighted that neuron and astrocyte-derived EVs decrease in the plasma following acute traumatic brain injury [41, 42]. Our results suggest that a similar effect may also be occurring with immune cell-derived EVs in the presence of elevated NfL and neuronal injury. Interestingly, the presence of NfL has also been shown to increase TNF production in PBMCs [43] and may perhaps help promote the migration of T-cell-derived EVs across the BBB. A similar result has been previously reported in mice following a systemic LPS injection [33].

While elevated pNfL levels are suggested as a measure of axonal injury in MS, we also investigated any relationship(s) between sub-populations of immune cell-derived EVs and MRI activity, clinical relapse history, and clinical disability progression (Fig. 4). Previous investigations have noted that EVs derived from neurons are smaller in MS patients during active relapse, yet larger in MRI-active patients compared to MRI-stable [26]. When investigating levels of specific immune cell-derived EV subsets, while we did not measure any differences in total plasma EV size (Fig. 4C), a cross-sectional analysis demonstrated that levels of CD4^+^ and CD8^+^ EVs were significantly increased in the plasma of active MS patients compared to clinically stable (Fig. 4D). These results are consistent with previous reports whereby the plasma concentration of T-cell-derived EVs was elevated in RRMS during a clinical relapse [26]. Our results suggest that an analysis of EVs from specific T-cell subsets provides a deeper understanding of the potential involvement of EVs during a clinical relapse. The increase in EVs from T-cell subsets may be the result of immune cell activation, as pro-inflammatory stimulants can increase the release of EVs from various cell types, including microglia, mesenchymal stem cells, and neutrophils [44–46]. Taken together, these data highlight the complex relationship between clinical versus radiological activity, which may be driven by different cellular and molecular mechanisms. A recent report has also emphasized the important temporal relationship between NfL levels and radiologic disease activity, whereby the majority of patients with Gd+ lesions do not have elevated NfL levels and suggests that the timing and type of radiologic activity in MS is critical when interpreting an NfL measurement [47].

In conclusion, we have demonstrated that plasma EVs sub-populations secreted from T cells are significantly altered in active RRMS patients and are associated with the rate of clinical disability, axonal injury, and disease activity. Importantly, changes in the numbers of immune cell subsets were not correlated with changes in levels of respective EV counterparts, further confirming that changes in EVs within plasma are likely representative of immune cell activity/status and not due to the overall abundance of individual immune cells within circulation [19]. Moving forward, measuring the size of specific immune cell-derived EVs may be suggestive of immune cell activation and serve as a biomarker in MS. Furthermore, given that DMTs are immunomodulatory and their use has previously been shown to influence the concentration of EVs in plasma [36, 48], future studies aimed at investigating EVs in the context of DMTs are warranted. The current study also highlights the importance of an intra-patient and longitudinal analysis when investigating EVs as potential biomarkers. Prior to this study, only cross-sectional data investigating EVs as biomarkers in MS has been published, which provides only a snapshot of EV sub-populations at one time-point and does not account for the inter-individual variability that needs to be accounted for when attempting to identify EVs as biomarkers in MS. The complex patterning of circulating EVs may also provide a better understanding of the events involved in MS pathophysiology and further elucidate the cellular/molecular mechanisms that influence disease progression in MS.

## Supplementary Material

uxaf003_suppl_Supplementary_Figure_S1

uxaf003_suppl_Supplementary_Figure_S2

uxaf003_suppl_Supplementary_Figure_S3

uxaf003_suppl_Supplementary_Figure_S4

uxaf003_suppl_Supplementary_Figure_S5

uxaf003_suppl_Supplementary_Figure_S6

uxaf003_suppl_Supplementary_Figure_S7-S8

uxaf003_suppl_Supplementary_Figure_S9

## Data Availability

The authors confirm that the data supporting the findings of this study are available in the Supporting Data Values file, which includes values underlying graphed data and reported means presented in both the main text and supplemental material.

## Abbreviations

CSF: cerebrospinal fluid
DMT: disease-modifying therapy
EDSS: expanded disability status scale
EVs: extracellular vesicles
HITMS: Health research innovation team in multiple sclerosis
MRI: magnetic resonance imaging
MS: multiple sclerosis
NEDA: no evidence of disease activity
NfL: neurofilament light chain
RRMS: relapsing remitting multiple sclerosis
