# Supplementary figures and images for: Investigating T-cell-derived extracellular vesicles as biomarkers of disease activity, axonal injury, and disability in multiple sclerosis
Source: Clin Exp Immunol. 2025 Jan 11;219(1):uxaf003. doi: 10.1093/cei/uxaf003 (PMC11791523; doi:10.1093/cei/uxaf003)

## Slide 1
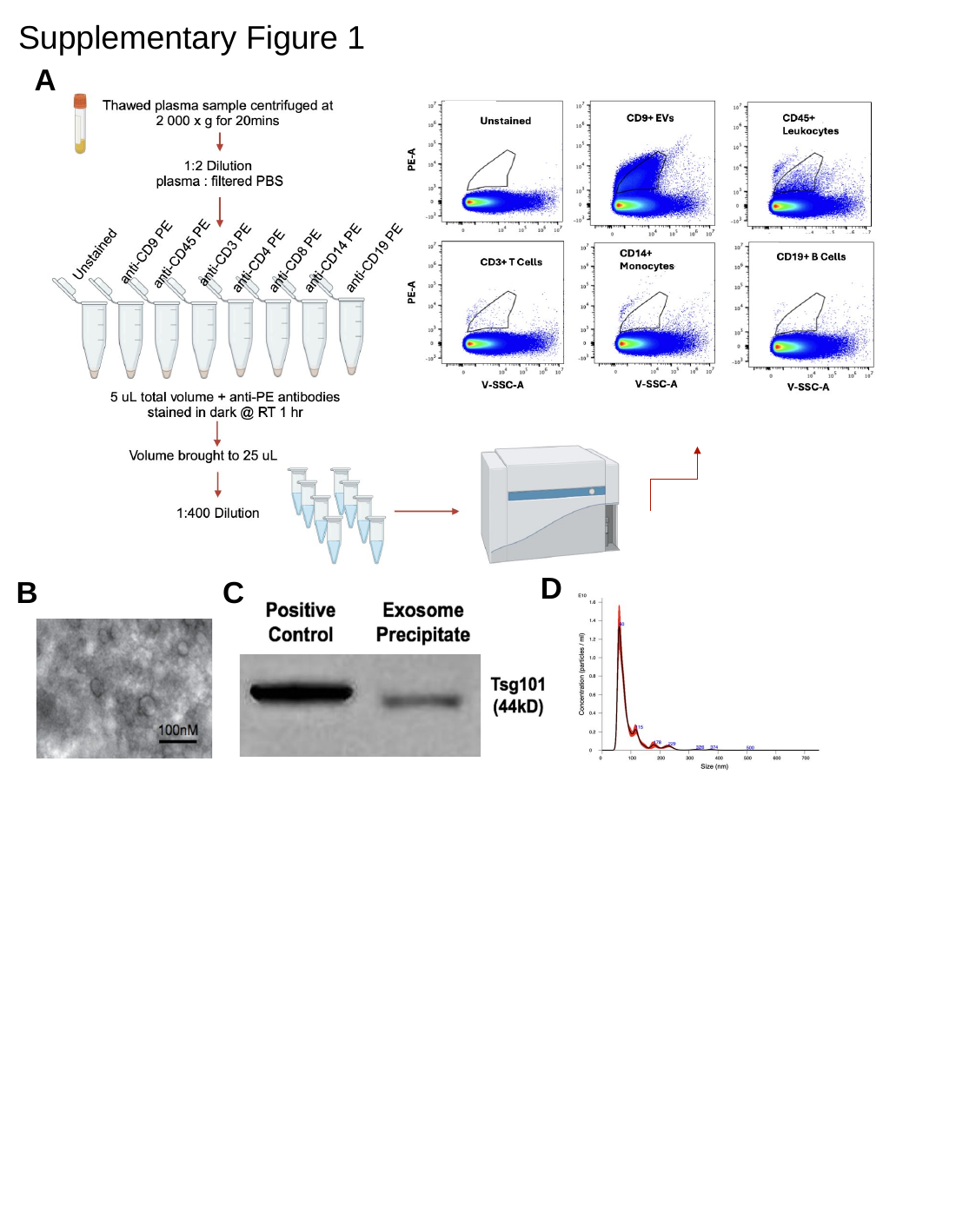

Supplementary Figure 1
A
A
E
D
B
C

Supplement: uxaf003_suppl_Supplementary_Figure_S1 [file uxaf003_suppl_Supplementary_Figure_S1.pptx]

## Slide 1
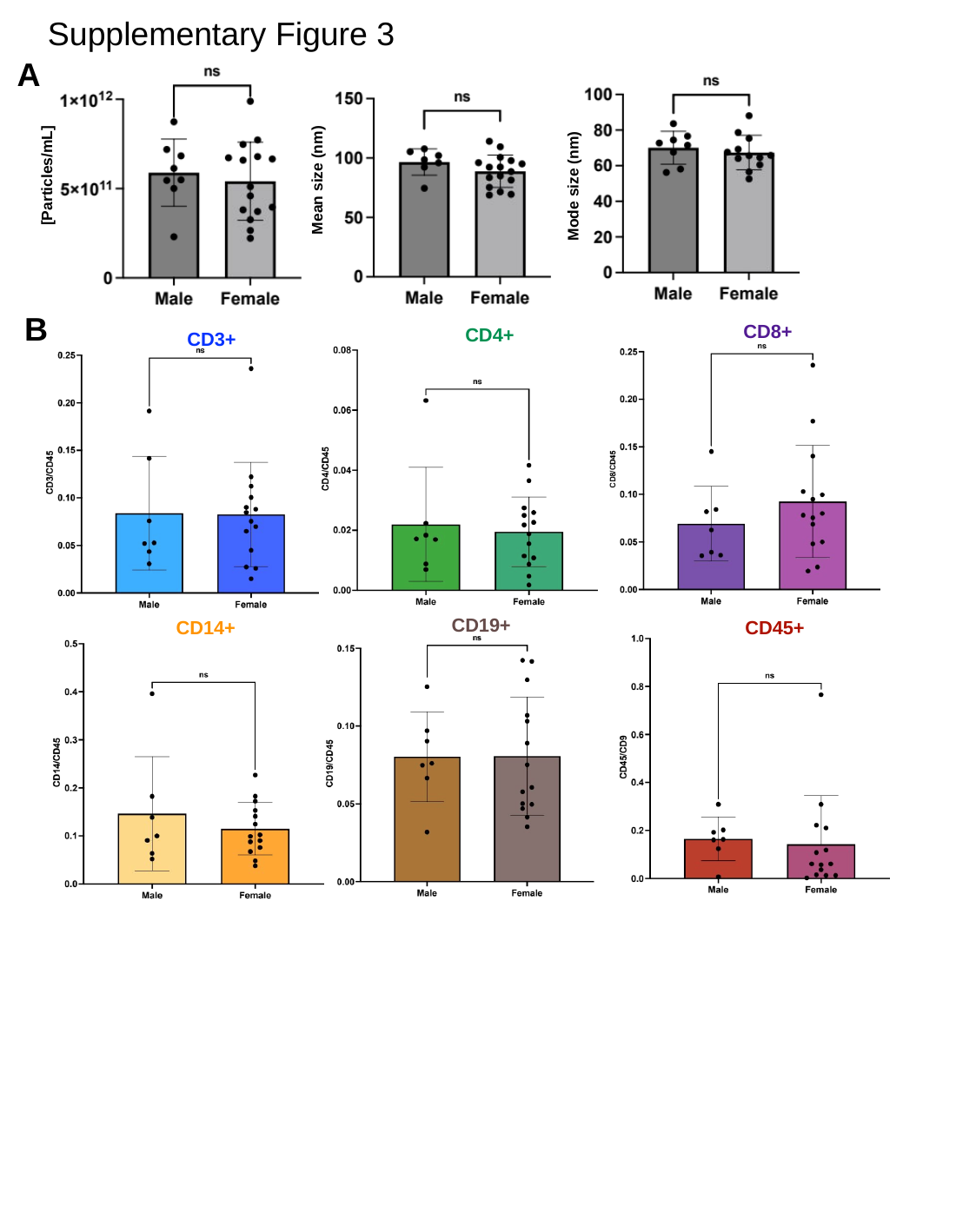

Supplementary Figure 3
A
 [Particles/mL]
Mean size (nm)
Mode size (nm)
B
CD8+
CD4+
CD3+
CD19+
CD14+
CD45+

Supplement: uxaf003_suppl_Supplementary_Figure_S3 [file uxaf003_suppl_Supplementary_Figure_S3.pptx]
