# Supplementary material for: Investigating T-cell-derived extracellular vesicles as biomarkers of disease activity, axonal injury, and disability in multiple sclerosis
Source: Clin Exp Immunol. 2025 Jan 11;219(1):uxaf003. doi: 10.1093/cei/uxaf003 (PMC11791523; doi:10.1093/cei/uxaf003)
Supplement: uxaf003_suppl_Supplementary_Figure_S2 [file uxaf003_suppl_Supplementary_Figure_S2.pptx]

## Slide 1
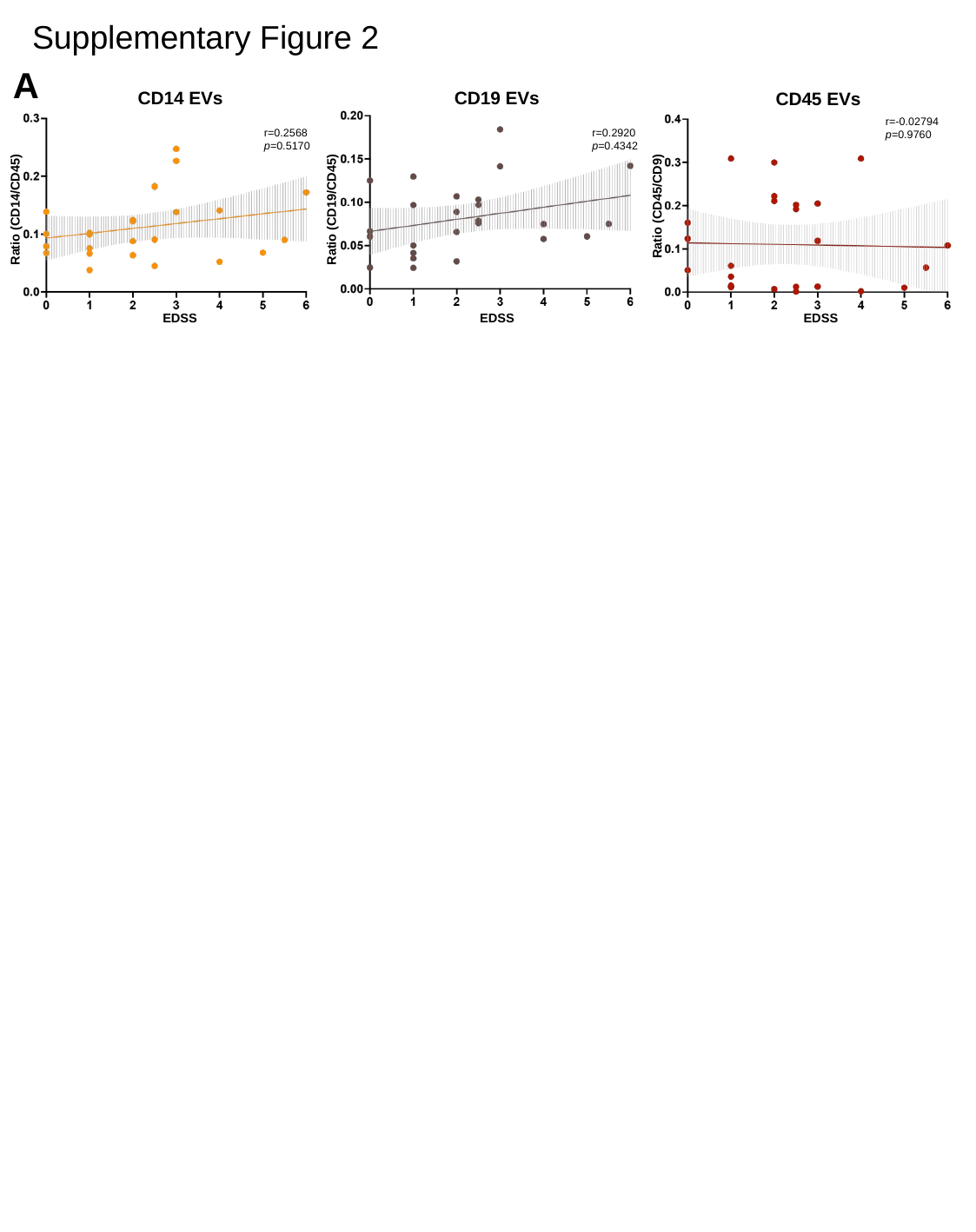

Supplementary Figure 2
A
CD14 EVs
CD19 EVs
CD45 EVs
r=-0.02794
p=0.9760
r=0.2568
p=0.5170
r=0.2920
p=0.4342
Ratio (CD45/CD9)
Ratio (CD19/CD45)
Ratio (CD14/CD45)
EDSS
EDSS
EDSS
