# Supplementary material for: Investigating T-cell-derived extracellular vesicles as biomarkers of disease activity, axonal injury, and disability in multiple sclerosis
Source: Clin Exp Immunol. 2025 Jan 11;219(1):uxaf003. doi: 10.1093/cei/uxaf003 (PMC11791523; doi:10.1093/cei/uxaf003)
Supplement: uxaf003_suppl_Supplementary_Figure_S4 [file uxaf003_suppl_Supplementary_Figure_S4.pptx]

## Slide 1
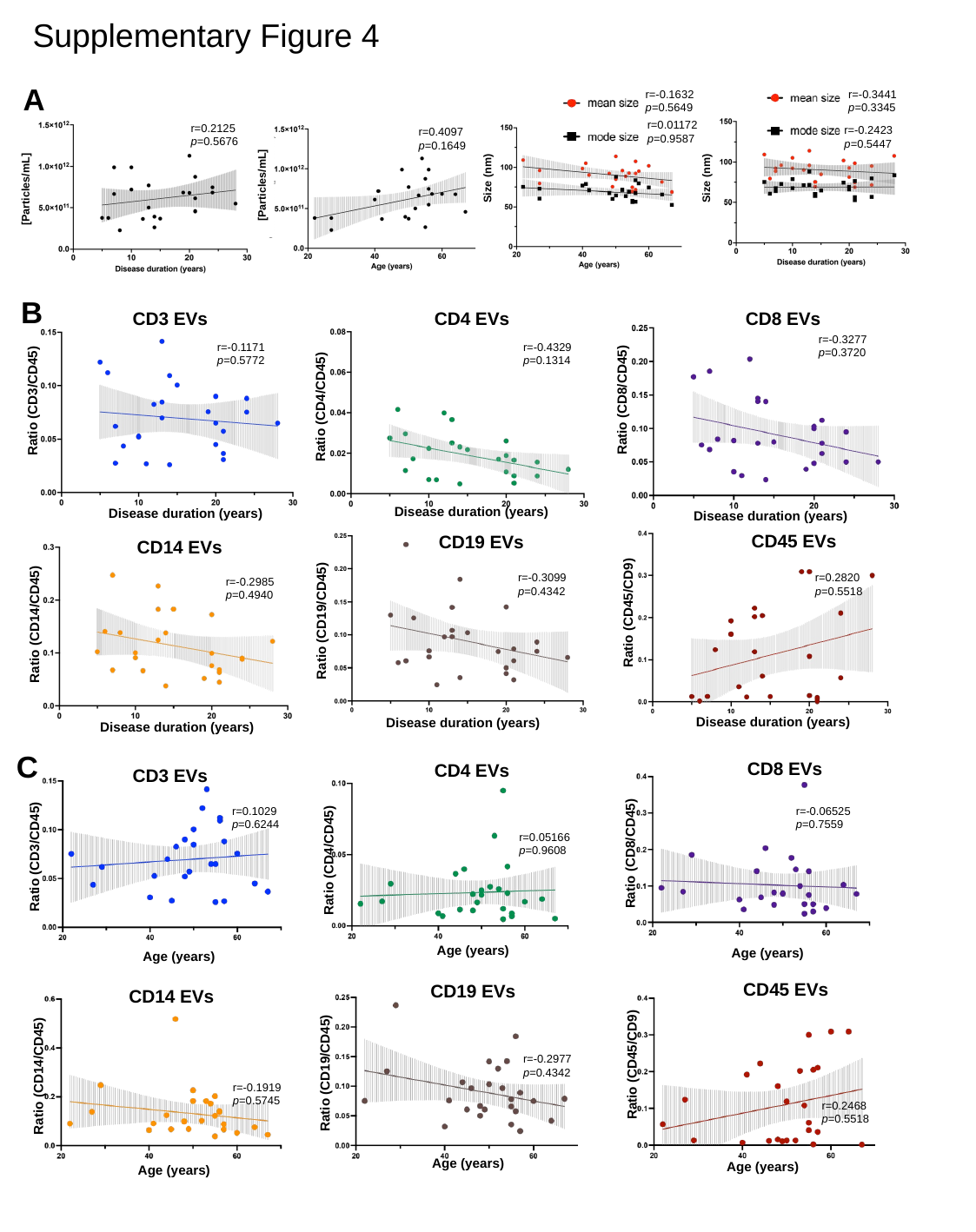

Supplementary Figure 4
A
r=-0.1632
p=0.5649
r=-0.3441
p=0.3345
r=0.01172
p=0.9587
r=0.2125
p=0.5676
r=-0.2423
p=0.5447
r=0.4097
p=0.1649
 [Particles/mL]
Size (nm)
 [Particles/mL]
Size (nm)
B
CD3 EVs
CD4 EVs
CD8 EVs
r=-0.3277
p=0.3720
r=-0.1171
p=0.5772
r=-0.4329
p=0.1314
Ratio (CD8/CD45)
Ratio (CD4/CD45)
Ratio (CD3/CD45)
Disease duration (years)
Disease duration (years)
Disease duration (years)
CD45 EVs
CD19 EVs
CD14 EVs
r=-0.3099
p=0.4342
r=0.2820
p=0.5518
r=-0.2985
p=0.4940
Ratio (CD45/CD9)
Ratio (CD19/CD45)
Ratio (CD14/CD45)
Disease duration (years)
Disease duration (years)
Disease duration (years)
C
CD8 EVs
CD4 EVs
CD3 EVs
r=0.1029
p=0.6244
r=-0.06525
p=0.7559
Ratio (CD8/CD45)
r=0.05166
p=0.9608
Ratio (CD4/CD45)
Ratio (CD3/CD45)
Age (years)
Age (years)
Age (years)
CD45 EVs
CD19 EVs
CD14 EVs
Ratio (CD45/CD9)
Ratio (CD19/CD45)
r=-0.2977
p=0.4342
Ratio (CD14/CD45)
r=-0.1919
p=0.5745
r=0.2468
p=0.5518
Age (years)
Age (years)
Age (years)
