# Supplementary material for: Investigating T-cell-derived extracellular vesicles as biomarkers of disease activity, axonal injury, and disability in multiple sclerosis
Source: Clin Exp Immunol. 2025 Jan 11;219(1):uxaf003. doi: 10.1093/cei/uxaf003 (PMC11791523; doi:10.1093/cei/uxaf003)
Supplement: uxaf003_suppl_Supplementary_Figure_S5 [file uxaf003_suppl_Supplementary_Figure_S5.pptx]

## Slide 1
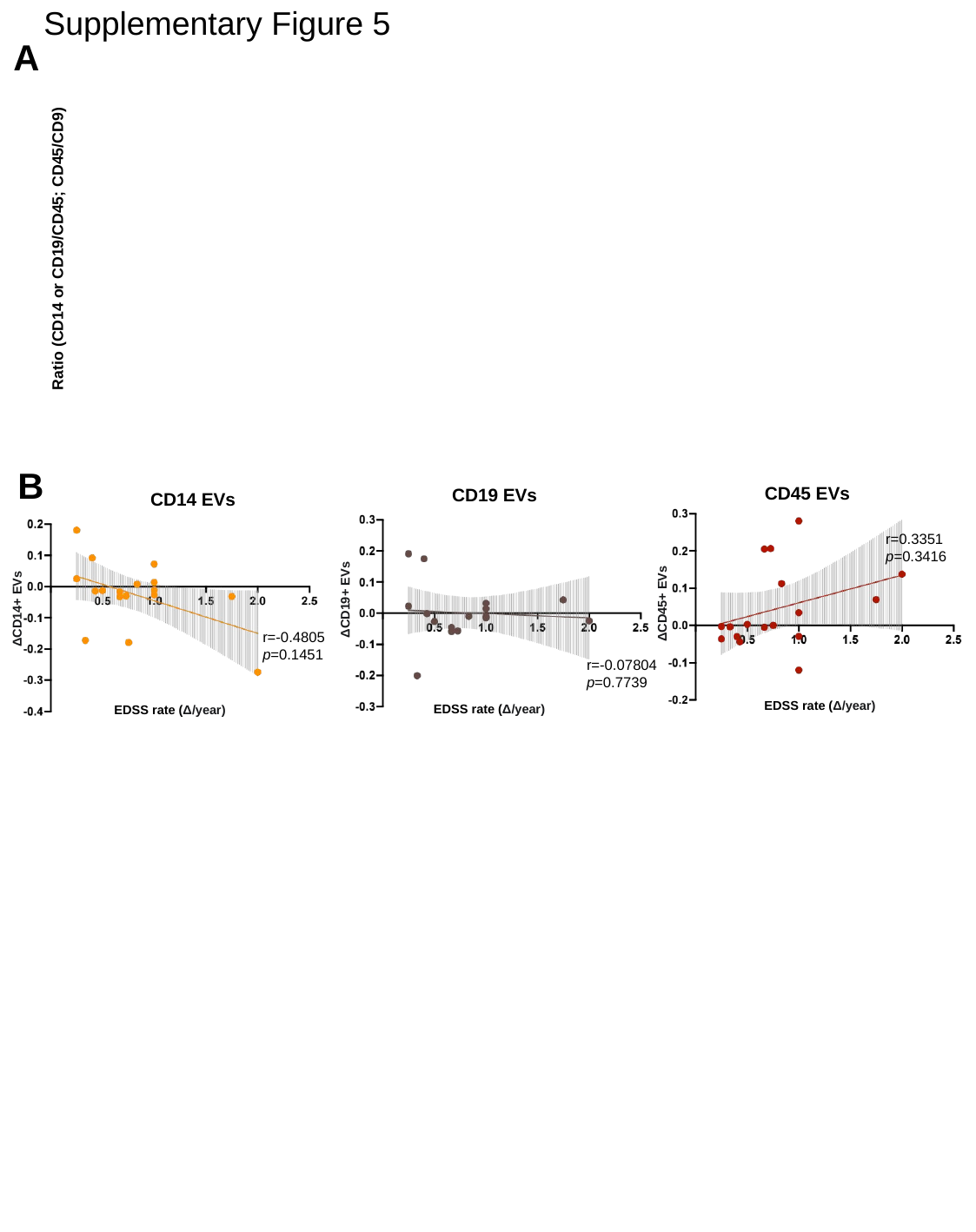

Supplementary Figure 5
A
Ratio (CD14 or CD19/CD45; CD45/CD9)
B
CD45 EVs
CD19 EVs
CD14 EVs
r=0.3351
p=0.3416
ΔCD45+ EVs
ΔCD19+ EVs
ΔCD14+ EVs
r=-0.4805
p=0.1451
r=-0.07804
p=0.7739
EDSS rate (Δ/year)
EDSS rate (Δ/year)
EDSS rate (Δ/year)
