# Supplementary material for: Investigating T-cell-derived extracellular vesicles as biomarkers of disease activity, axonal injury, and disability in multiple sclerosis
Source: Clin Exp Immunol. 2025 Jan 11;219(1):uxaf003. doi: 10.1093/cei/uxaf003 (PMC11791523; doi:10.1093/cei/uxaf003)
Supplement: uxaf003_suppl_Supplementary_Figure_S6 [file uxaf003_suppl_Supplementary_Figure_S6.pptx]

## Slide 1
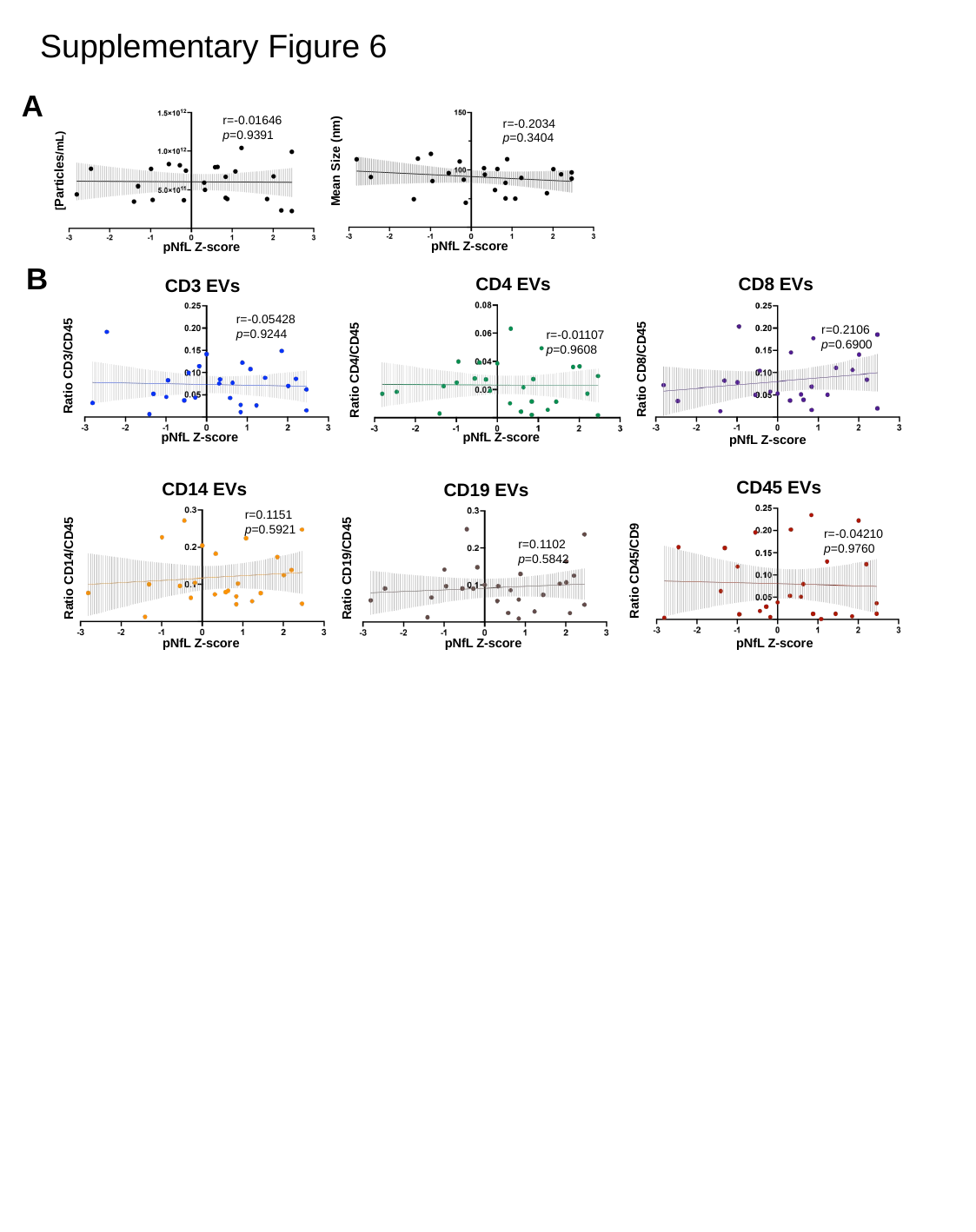

Supplementary Figure 6
A
r=-0.01646
p=0.9391
r=-0.2034
p=0.3404
Mean Size (nm)
[Particles/mL)
pNfL Z-score
pNfL Z-score
B
CD4 EVs
CD8 EVs
CD3 EVs
r=-0.05428
p=0.9244
r=0.2106
p=0.6900
r=-0.01107
p=0.9608
Ratio CD3/CD45
Ratio CD8/CD45
Ratio CD4/CD45
pNfL Z-score
pNfL Z-score
pNfL Z-score
CD45 EVs
CD14 EVs
CD19 EVs
r=0.1151
p=0.5921
r=-0.04210
p=0.9760
r=0.1102
p=0.5842
Ratio CD14/CD45
Ratio CD19/CD45
Ratio CD45/CD9
pNfL Z-score
pNfL Z-score
pNfL Z-score
