# Supplementary material for: Investigating T-cell-derived extracellular vesicles as biomarkers of disease activity, axonal injury, and disability in multiple sclerosis
Source: Clin Exp Immunol. 2025 Jan 11;219(1):uxaf003. doi: 10.1093/cei/uxaf003 (PMC11791523; doi:10.1093/cei/uxaf003)
Supplement: uxaf003_suppl_Supplementary_Figure_S7-S8 [file uxaf003_suppl_Supplementary_Figure_S7-S8.pptx]

## Slide 1
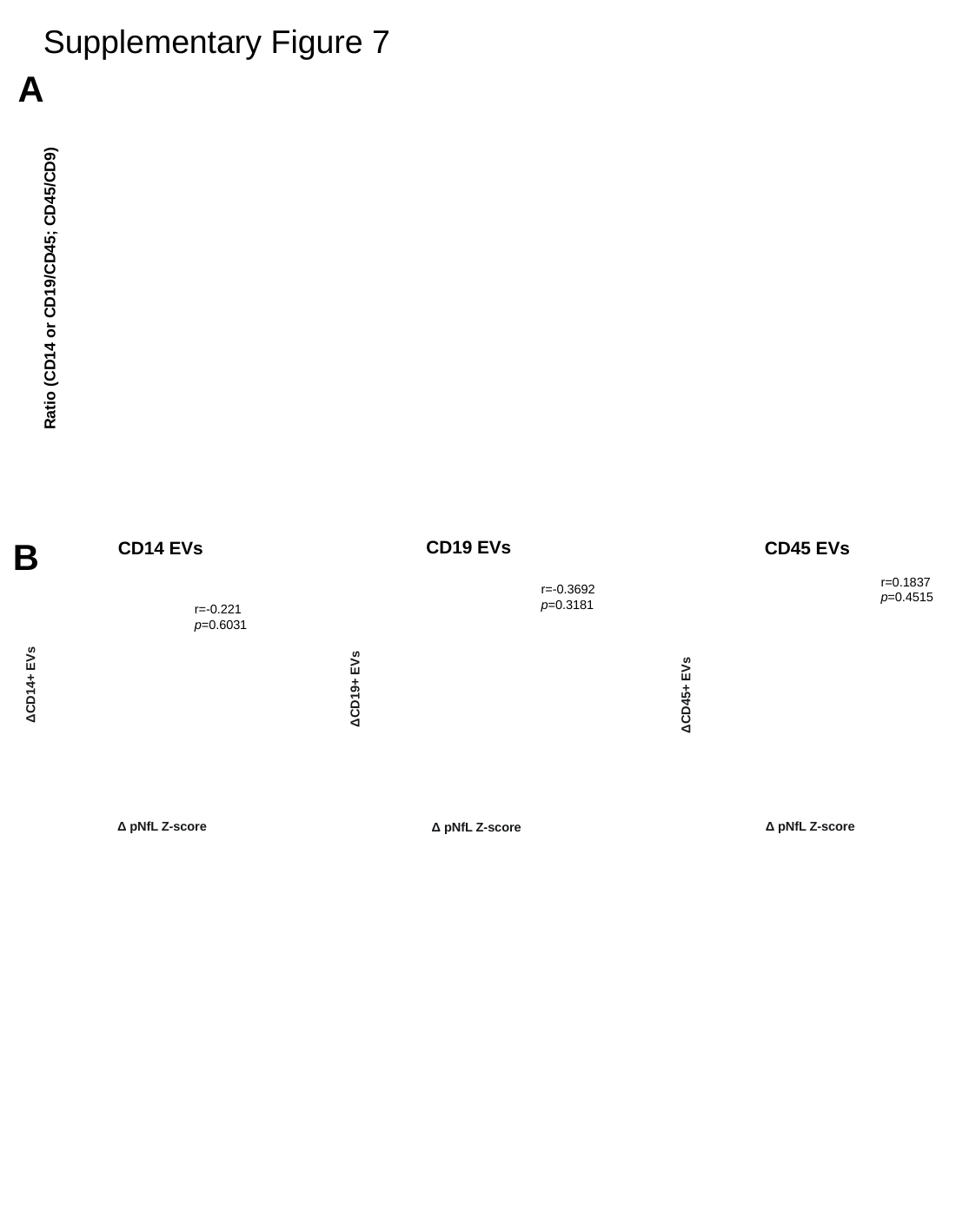

Supplementary Figure 7
A
Ratio (CD14 or CD19/CD45; CD45/CD9)
B
CD19 EVs
CD45 EVs
CD14 EVs
r=0.1837
p=0.4515
r=-0.3692
p=0.3181
r=-0.221
p=0.6031
ΔCD14+ EVs
ΔCD45+ EVs
ΔCD19+ EVs
Δ pNfL Z-score
Δ pNfL Z-score
Δ pNfL Z-score
