# Supplementary material for: Investigating T-cell-derived extracellular vesicles as biomarkers of disease activity, axonal injury, and disability in multiple sclerosis
Source: Clin Exp Immunol. 2025 Jan 11;219(1):uxaf003. doi: 10.1093/cei/uxaf003 (PMC11791523; doi:10.1093/cei/uxaf003)
Supplement: uxaf003_suppl_Supplementary_Figure_S9 [file uxaf003_suppl_Supplementary_Figure_S9.pptx]

## Slide 1
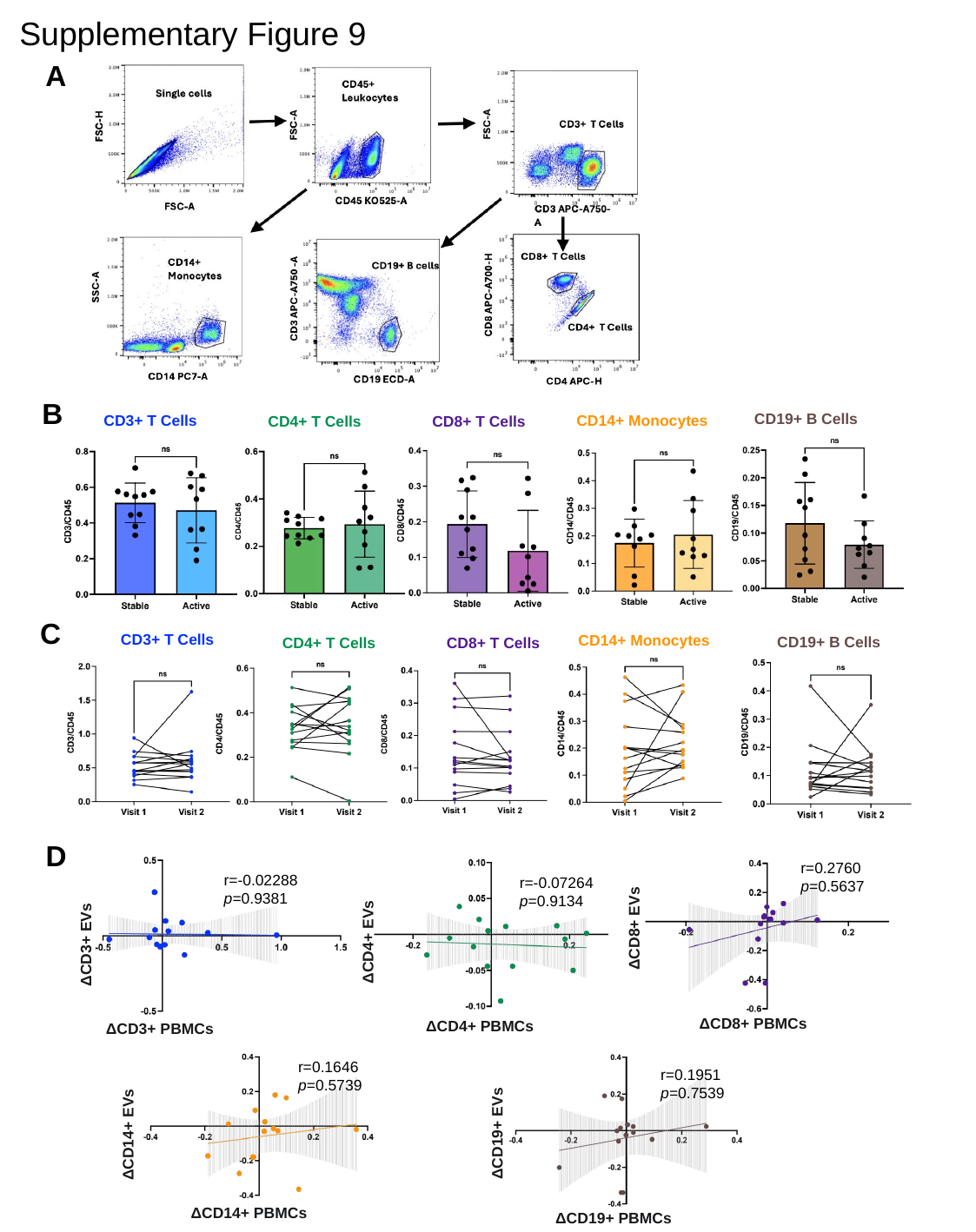

Supplementary Figure 9
A
B
CD19+ B Cells
CD14+ Monocytes
CD3+ T Cells
CD4+ T Cells
CD8+ T Cells
C
CD3+ T Cells
CD14+ Monocytes
CD19+ B Cells
CD4+ T Cells
CD8+ T Cells
D
r=0.2760
p=0.5637
r=-0.02288
p=0.9381
r=-0.07264
p=0.9134
ΔCD8+ EVs
ΔCD4+ EVs
ΔCD3+ EVs
ΔCD8+ PBMCs
ΔCD4+ PBMCs
ΔCD3+ PBMCs
r=0.1646
p=0.5739
r=0.1951
p=0.7539
ΔCD19+ EVs
ΔCD14+ EVs
ΔCD14+ PBMCs
ΔCD19+ PBMCs
